# Scenario drafting for early technology assessment of next generation sequencing in clinical oncology

**DOI:** 10.1186/s12885-016-2100-0

**Published:** 2016-02-06

**Authors:** S.E.P. Joosten, V.P. Retèl, V.M.H. Coupé, M.M. van den Heuvel, W.H. van Harten

**Affiliations:** Department of Clinical Epidemiology and Biostatistics, VU University Medical Centre Amsterdam, 1081 HZ Amsterdam, The Netherlands; Department of Psychosocial Research and Epidemiology, Netherlands Cancer Institute-Antoni van Leeuwenhoek Hospital, 1066 CX Amsterdam, The Netherlands; Department of Thoracic Oncology, Netherlands Cancer Institute-Antoni van Leeuwenhoek Hospital, 1066 CX Amsterdam, The Netherlands; School of Governance and Management, University of Twente, MB-HTSR, PO Box 217, 7500 AE Enschede, The Netherlands

**Keywords:** Health Technology Assessment, Next Generation Sequencing, Clinical oncology, Personalized, Challenges

## Abstract

**Background:**

Next Generation Sequencing (NGS) is expected to lift molecular diagnostics in clinical oncology to the next level. It enables simultaneous identification of mutations in a patient tumor, after which targeted therapy may be assigned. This approach could improve patient survival and/or assist in controlling healthcare costs by offering expensive treatment to only those likely to benefit. However, NGS has yet to make its way into the clinic. Health Technology Assessment can support the adoption and implementation of a novel technology, but at this early stage many of the required variables are still unknown.

**Methods:**

Scenario drafting and expert elicitation via a questionnaire were used to identify factors that may act as a barrier or facilitate adoption of NGS-based molecular diagnostics. Attention was paid to predominantly elicit quantitative answers, allowing their use in future modelling of cost-effectiveness.

**Results:**

Adequately informing patients and physicians, the latters’ opinion on clinical utility and underlying evidence as well as presenting sequencing results within a relevant timeframe may act as pivotal facilitators. Reimbursement for NGS-based testing and accompanying therapies (both general and in case of off-label prescription) was found to be a potential barrier. Competition on the market and demonstrating clinical utility may also be challenging. Importantly, numerous quantitative values for variables related to each of these potential barriers/facilitators, such as such as desired panel characteristics, willingness to pay or the expected number of targets identified per person, were also elicited.

**Conclusions:**

We have identified several factors that may either pose a barrier or facilitate the adoption of NGS in the clinic. We believe acting upon these findings, for instance by organizing educational events, advocating new ways of evidence generation and steering towards the most cost-effective solution, will accelerate the route from bench-to-bedside. Moreover, due to the methodology of expert elicitation, this study provides parameters that can be incorporated in future cost-effectiveness modeling to steer the development of NGS gene panels towards the most optimal direction.

**Electronic supplementary material:**

The online version of this article (doi:10.1186/s12885-016-2100-0) contains supplementary material, which is available to authorized users.

## Background

In recent years, the cost and time required for large scale sequencing have rapidly decreased, catalyzing an increased understanding of genetic variation in both health and disease. Relatively cheap next generation sequencing (NGS) may confer great benefit in a clinical setting as well, especially in oncology [1]. Many institutes are currently developing NGS-based gene panels, which investigate the presence of multiple mutations in a single tumor at once. Subsequently, a specific targeted therapy may be assigned thereby potentially improving clinical outcome [2]. While many experts advocate that simultaneous testing of genes also has the potential to be more cost-effective than performing sequential single-gene assays, this has yet to be shown [3]. We define a NGS gene panel as “a multiplex predictive test which explores limited regions of tumor DNA/RNA for aberrations that can be used as a molecular target for therapy”.

Meanwhile, NGS has reached the molecular diagnostic market and is expected to slowly replace single-gene molecular diagnostic tests [4]. Currently, within the Netherlands, hospitals have started with the implementation of NGS for diagnostics, using techniques ranging from single gene testing to small, medium or large NGS panels. Beyond biology, the adoption of NGS for large scale molecular diagnostics will also depend on a variety of organizational, societal and economic factors [5, 6]. For instance, will hospitals be able to supply tissue meeting NGS requirements? Are physicians up to date on pharmacogenomics to use such a test in the clinic? And importantly, can society afford personalized medicine at all, given the costs associated with sequencing as well as extremely expensive targeted [5, 6]? Due to the increased pressure to control ever-rising healthcare costs, reliable input regarding novel technologies on these factors is becoming increasingly more important. To our knowledge there are as yet no widely accepted national policies on NGS-based panels apart from the French initiative to centralize services for a specified number of molecular tests in regional centers under the Institut National du Cancer (INCa) umbrella.

A commonly used methodology to estimate and evaluate the impact of a novel technology is Health Technology Assessment (HTA), which is increasingly being used to support policy and reimbursement decisions regarding medical interventions [7]. Early stage TA can help to expedite further development and guide the adoption of a promising technology in the clinic [8]. We have previously performed such an early TA assessment for the introduction and adoption of a 70-gene prognosis-signature for breast cancer [9, 10]. As part of this assessment, to fill in evidence gaps in cost-effectiveness analysis, we used scenario drafting as originally developed by Royal Dutch Shell. By describing potential directions of development, Shell is able to anticipate events possibly affecting their market position and timely adapt corporate strategy [11–13]. In case of the 70-gene array, we drafted several scenarios that represented likely patterns of its diffusion across the health care system focusing on features that were still likely to change during development, such as clinical, economic, patient-related, and organizational parameters [10]. Some of these were subsequently incorporated into a cost-effectiveness analysis [14].

In this paper, we report on scenario drafting concerning the adoption and implementation of NGS gene panels in clinical oncology among professionals. Our objective was first; to identify critical barriers and facilitators that may affect the speed of adoption of such panels in clinical practice and second; to estimate values of quantitative parameters for future cost-effectiveness modeling.

## Methods

### Background research

**Fig. 1:**
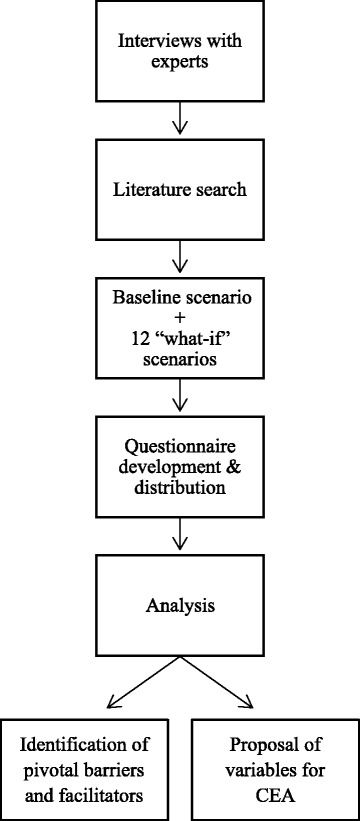
Overview of methodology. CEA = Cost-effectiveness analysis

We first interviewed in-house experts (Netherlands Cancer Institute) specialized in (molecular) diagnostics, patient management and/or next-generation sequencing to identify variables that are likely to affect the speed of adoption of NGS-panels (Fig. 1). More info was gathered using Pubmed and Google Scholar, by searching for recent papers using (combinations of) the terms “cancer”/”oncology”/”tumor”/”clinical” + “personalized medicine”, “precision medicine”, “genomic medicine”, “stratified medicine”, “targeted therapy”, “tailored therapy”, “pharmacogenomics”, “next-generation sequencing”, “capture-based sequencing”, “multiplex sequencing”, “molecular diagnostics”, “companion diagnostics”, “genetic testing”, “predictive biomarkers”, “economics”, “cost-effectiveness”, “perspectives”, “costs”, “implementation”, “challenges”, “reimbursement”, “storage”, “data”, “patients”, “physicians”. This resulted in thousands of papers often discussing the same topics. We selected papers for our background research that discussed multiple issues surrounding adoption and implementation of NGS simultaneously, highlighted the perspective of several stakeholders, were written in English and published no longer than 10 years ago, resulting in a set of 106 papers.

### Scenario drafting

Using all the gathered background information, we drafted one baseline scenario describing the diffusion of NGS gene panels for personalized cancer treatment in general and twelve “what if” scenario deviations, which represent developments that may positively or negatively affect the speed of diffusion. Next, we drafted a questionnaire to elicit expert opinion on the specifics and likelihood of our “what-if” scenarios.

### Questionnaire construction & distribution

First, forty-one questions were specifically designed to elicit quantitative answers in order to use our data in future cost-effectiveness modeling. Since we are planning to perform such modeling for at least colorectal cancer (CRC), non-small cell lung cancer (NSCLC) and melanoma, we often posed questions for each patient population separately. Input from Netherlands Cancer Institute (NKI) employees was used to prevent ambiguity in language.

Next, subsets of questions were used to construct three questionnaires (Additional file 1), each one specifically tailored to the expertise of physicians, biologists or policy workers. All versions were accompanied by the same cover letter providing background on NGS gene panels and explaining the purpose of our research. At the end, respondents were asked to rate the likelihood of the twelve scenarios on a scale from 0-100 %.

The questionnaires were distributed via email to a sample of NKI employees and external (partly international) stakeholders. Given the complexity of NGS-based diagnostics and in view of the very early stage of development and uncertainty surrounding clinical utility, we decided to focus on technical experts and clinicians first. External recipients had been in previous contact with the hospital or were selected because of published work on related matters. After a week, a reminder was sent to non-responders.

### Data collection and analysis

A database of respondents answers was created using Adobe Acrobat X Pro and variation among expert opinions was assessed visually using colored 2D-dotplots as well as by descriptive statistics using IBM SPSS statistics. If answers were illegible these were excluded from our analysis and we also assessed whether (missing) values could be attributed to a certain respondent subgroup (e.g. profession, specialization, internal/external).

### Consent statement

This study was made possible by elicitation of expert’ opinion via a questionnaire. The procedure was verified with the protocol review committee. Participation was on voluntary basis and filled-in questionnaires were anonymized prior to analysis. Upon invitation, experts were informed that their answers would be used anonymously to improve scenarios. No patients nor children, parents or guardians were involved.

### Ethics statement

According to institutional guidelines, it was verified with the protocol review committee that no ethical review nor consent was needed for this study.

## Results

Questions posed in our survey often relate to several scenarios simultaneously, therefore we have clustered results into the following domains: social factors; technical factors; market access; clinical utility & evidence generation and reimbursement. For every domain we first report the estimated likelihood of scenario occurrence in percentages (Table 2) and next we discuss our findings on associated parameters (Table 3) and their relevance for cost effectiveness modeling. Summarizing these findings, we have labeled a scenario as a potential facilitator or barrier. All scores are represented as mean results ± standard deviation. In some cases absolute numbers are mentioned to depict clear disagreement among respondents.

### Respondent characteristics

In total, 29 questionnaires were completed by 14 physicians (specialized in general oncology, pulmonology, dermatology or pathology), 11 biologists (research and diagnostics), three policy workers and one epidemiologist; 12 from within and 17 from outside the NKI (Table 1).

**Table 1:** Respondent characteristics

Profession		Respondents (n)	
		NKI	External
Physicians	*Oncologist*	5	4
	*Other* ^a^	4	1
Biologist	*Research*	2	5
	*Diagnostics*	0	4
Policy		0	3
Epidemiologist		1	0
Total		12	17

Respondent specifics are described here, distinguishing between Netherlands Cancer Institute employees (NKI) and external respondents. 4 respondents were situated outside the Netherlands.^a^ Other: specializations beyond medical oncology included pathology (2), pulmonology (1), dermatology (1) or surgery (1)

### Social factors

The likelihood of scenario 1 occurrence within 5 years, in which patients will be interested in NGS panels and will demand lots of information on this molecular approach was estimated at 66,5 % (±28,1) (Table 2).

**Table 2:** Baseline-and what-if scenarios

Baseline scenario: “Within 5–10 years, NGS gene panels will become common practice for personalized treatment in oncology”			
Domain	What-if scenarios (likelihood ± SD)	Effect	Barrier/facilitator
Social	1. *Patient perspective* (*66*,*5* ± *28*,*1*; *n* = *13*) Patients will demand lots of information on NGS-based panels, but will nevertheless be very interested in using them.	Higher uptake and more compliance	*Pivotal facilitators*
	2. *Medical professional perspective* (*16*,*5* ± *8*,*8*; *n* = *10 and 64*,*0* ± *8*,*9*; *n* = *5*) Medical professionals remain unconvinced of the clinical benefit that can be gained using NGS-panels and targeted therapy.		
Technical	3. *Organization* (*84*,*4* ± *18*,*5*; *n* = *25*) The time required for preparation, NGS and analysis of a biopsy will decrease so that patients will receive results within ten days after biopsy.	Higher uptake and less failures	
	4. *FF* versus *FFPE* (*86*,*8* ± *13*,*1*; *n* = *11 and 16*,*2* ± *9*,*4*; *n* = *13*) If reliable sequencing results can only be obtained by using FF tissue, the use of NGS-based panels will remain limited.		
Reimbursement	5. *Reimbursement* (*40*,*3* ± *24*,*1*; *n* = *18*) A ‘minimal requirements’ agreement between institutes developing NGS-based gene panels has resulted in national reimbursement policy of such panels.	Less uptake	*Pivotal barriers*
Clinical utility and evidence generation	6. *Clinical Utility* (*50*,*4* ± *31*,*4*; *n* = *25*) Demonstrating clinical utility of NGS-panels will take at least a couple more years, adoption of this technology will only succeed once that point is reached.	No improved survival and slow release of new target/therapy combinations	
	7. *Actionable targets* (*55*,*2* ± *23*,*7*; *n* = *26*) The number of mutations identified by NGS panels that can actually be targeted by therapy, remains limited.		
	8. *Off*-*label prescription* (*49*,*2* ± *31*,*1*; *n* = *18*) The medical community becomes more lenient towards off-label treatment.		
	9. *Revised evidence generation* (*65*,*5* ± *27*,*9*; *n* = *29*) Evidence from less time-consuming clinical studies than RCT III, will be considered valid to include new targets in NGS-based gene panels		
Market access	10. *Competition from a different field* (*45*,*0* ± *21*,*7*; *n* = *25*) Another type of technology enters the Dutch healthcare, decreasing the popularity of NGS-based gene panels.	Less uptake	
	11. *Competition within the field* (*64*,*2* ± *21*,*9*; *n* = *12*) Another NGS-based panel outcompetes the NKI-panel, regardless of its additional features.		
	12. *Intellectual property* (*45*,*6* ± *28*,*7*; *n* = *25*) Competitors offering NGS-based panels will be reluctant to share new biological insights generated by NGS-panels with each other, thereby decelerating the improvement of clinical utility for patients.		

Twelve potential deviations from a baseline scenario in which NGS-based gene panels are implemented in clinical oncology. Respondents were asked to rate the likelihood of their occurrence on a scale from 0-100 %. Since several scenarios were presented to relevant professions only, combined with some missing values, the number of respondents per scenario varied

When presented questions related to this “what-if” scenario, clinicians among respondents estimated that this will entail 65,3 % (±32,1) of patients suffering from metastatic cancer, compared to 28,3 % (±29,2) of patients with non-metastasized disease (Table 3). Respondents whom themselves are skeptical about using NGS for adjuvant treatment, estimated larger differences between the former two patient groups (Additional file 2). Additionally, they felt that 78,2 % (±16,1) and 41,5 % (±26,6) of those patients respectively would be willing to enroll in a clinical trial on a new targeted therapy. The number of extra minutes during a patient’s first consult to adequately inform them about NGS-based genetic testing was thought to lie around 13,2 (±12,4) minutes. Physicians themselves likely require extra education on NGS as well, estimated at 25,1 (±26,1) hours.

**Table 3:** Parameters and corresponding questions

Domain	Parameter		Q	Average ± SD
Social factors	Patients interested in NGS (prim/meta)		Q1	28,3 ± 29,2/65,3 ± 32,1
	Patients interested in trial (prim/meta)		Q2	41,5 ± 26,6/78,2 ± 16,1
	Consult extra (min)		Q3	13,2 ± 12,4
	Education extra (hrs.)		Q10	25,1 ± 26,1
	NGS Adoption given:	RCT3	Q6	84,9 ± 23,6
		Pros. observational		62,3 ± 20,3
		Retro. observational		39,6 ± 22,6
		Lower levels		16,7 ± 8,6
Technical Factors	Max. turnover rate (days)		Q9	17,8 ± 21,3
	Dutch institutes able to supply FF		Q16	50,5 ± 36,5
	Min. sensitivity/specificity		Q15	90,5 ± 5,7/89,0 ± 9,7
	Max. failure rate		Q17	18,4 ± 20,1
	Re-biopsy decline:	CRC	Q18	33,3 ± 23,6
		NSCLC		30,0 ± 22,9
		Melanoma		8,5 ± 5,9
	Re-biopsy unfeasible:	CRC	Q19	19,2 ± 11,1
		NSCLC		22,9 ± 16,0
		Melanoma		9,3 ± 10,0
	Min. storage tissue (yrs.)		Q14	24,2 ± 22,1
	Min. storage NGS results (yrs.)		Q13	22,6 ± 21,4
Reimbursement	Pay extra for NGS panel (euro)		Q12	380,8 ± 316,6
	Probability opt for NGS panel if €1000		Q40	44,0 ± 44,0
Clinical utility and evidence generation	Nb. Targets per patient		Q37	6,6 ± 7,5
	Nb. new therapies in five years		Q29	22,5 ± 20,4
	Off-label therapy required		Q22	30,2 ± 26,5
	Physicians willing to prescribe off-label		Q23	44,6 ± 31,6
	Probability reimbursement off-label		Q27	28,0 ± 32,0
	Lenient towards off-label (yrs.)		Q26	9,8 ± 12,4
Market access	Min. years NGS common practice		Q33	6,5 ± 6,3
	Min. years competition other technology		Q35	9,6 ± 5,5

Depicted are the mean results (percentages unless stated otherwise) and standard deviations on quantitative parameters, in order of appearance. Column Q refers to the number of the corresponding questions in the questionnaire (Additional file 1). *NGS* next generation sequencing, *Prim* primary cancer, *Meta* metastatic cancer, *FF* fresh frozen [tissue preservation]

Scenario 2, also related to social acceptability, described a situation in which physicians will remain unconvinced of the clinical benefit of large scale sequencing. The majority of respondents (10 out of 15) believed this scenario has a 16,5 % (±8,8) chance of occurring, while some (5/15) believed 64,0 % (±8,9) to be a more realistic number (Table 2).

Detailed questions on scenario 2 revealed that respondents estimate 84,9 % (±23,6) of physicians to adopt NGS gene panels if such panels have at least been validated by a phase 3 randomized controlled trial (Table 3). However, estimates of adoption decline in case of lower level studies (e.g. 62,3 % ±20,3 for a prospective observational study; 39,6 % ±22,6 for a retrospective observational study; 16,7 % ±8,6 for lower levels of evidence). 39 % of respondents themselves believe that NGS gene panels should only be offered to patients with advanced disease, but an equal percentage believe NGS should already be offered as an adjuvant solution. Others (22 %) found level of evidence,–toxicity and-benefit the most important indicators for use, irrespective of stage.

### Technical factors

Respondents rated the likelihood of scenario 3, describing a maximum of ten days turn-over time from biopsy to results, at 84,4 % (±18,5). When asked for their own preferences, a maximum of 17,8 (±21.3) days was indicated (Table 2).

NGS gene panels can vary in all kind of characteristics, including what type of tissue preservation (formalin-fixed paraffin embedded-FFPE-or fresh frozen-FF-biopsies) they can handle. When presented with scenario 4, in which only FF tissue would be able to generate reliable sequencing results, some respondents (11 out of 24) estimated a 86,8 % (±13,1) chance that this requirement will limit NGS adoption by medical professionals. Others (13 out of 24) found scenario 4 less likely to occur and estimated its likeliness to occur at 16,2 % (±9,4). This distinction was also found among physicians alone: 8 out of 11 estimated a 83,8 % (±19,2) chance of scenario occurrence, while the remaining opted for a 14,0 % (±8,9) chance.

An important parameter for scenario 4, the percentage of Dutch institutes capable of supplying FF biopsies, was estimated at 50,5 % (±36,5) (Table 3). Other questions into technical parameters that may affect both scenarios 3 and 4 revealed preferences for a minimal sensitivity/specificity of 90,5 % (±5,7) and 89,0 % (±9,7) respectively and a maximally acceptable failure rate (e.g. in case of too little DNA available) of 18,4 % (±20,1). If re-biopsy would be required to obtain reliable results, respondents expect a percentage of patients to decline (eg. 33,3 % ±23,6 in CRC; 30,0 % ±22,9 in NSCLC; 8,5 % ±5,9 in melanoma). Re-biopsy may even be unfeasible in a certain number of cases (19,2 % ±11,1 in CRC; 22,9 % ±16,0 in NSCLC; 9,3 % ±10,0 in melanoma). Respondents believed that residual tissue should be stored for minimally 24,2 (±22,1) years. For future reference, sequencing results carrying information beyond the scope of current treatment should be kept for at least 22,6 years (±21,4).

### Reimbursement

Scenario 5 depicts a situation where opposing institutes draw up a “minimal requirements” agreement on NGS gene panels and consequently a national reimbursement policy is implemented. Likelihood of scenario occurrence was estimated at 40,3 % (±24,1) (Table 2).

Addressing a related parameter, respondents felt that NGS-panel-compared to single gene diagnostics would justify €380,8 (±316,6) additionally (Table 3). The probability to opt for an FFPE-based NGS panel if priced at €1000 averaged at 44 % (±44,0).

### Clinical utility & evidence generation

Scenario 6, in which the route to demonstrating clinical utility still requires several years, was assessed 50,4 % (±31,4) likely. Scenario 7 described a theoretical limiting factor to that route, in which the number of therapeutic targets identified by NGS panels would remain limited. The chance of this scenario occurring was estimated at 55,2 % ±23,7 (Table 2).

Related to these scenarios, several parameters estimates were elicited (Table 3). Presented with the characteristics of the NKI-panel, respondents would expect to find 6.6 (±7,5) potential targets per patient. Also, they believe that in the following 5 years, 22,5 (±20,4) novel and approved targeted therapies (for new targets) will hit the market. Furthermore, they estimated that in 30,2 % (±26,5) of cases using NGS, a target for which off-label treatment is available will be identified.

Scenario 8, in which the medical community would become more lenient towards prescribing off-label medication, was found 49,2 % (±31,1) likely to occur (Table 2).

Under circumstances as described above, respondents expect 44,6 % (±31,6) of physicians to be willing to prescribe off-label therapy (regardless of reimbursement) (Table 3). The propensity of respondents themselves to do so varied given the level of evidence supporting a target/therapy combination and stage of disease (Table 4). They felt that the chance of reimbursement for off-label treatment is low (28,0 % ±0,32) and that the medical community will minimally need 9,8 (±12,4) years to become more lenient towards it.

**Table 4:** Respondent propensity to prescribe off-label therapy

Level of evidence	Adjuvant (n = 16)	Metastatic (n = 26)
*At least validated by an RCT3 in another type of cancer and an observational study for the type cancer you intent to treat*	6/16; 37,5 %	7/26; 27,0 %
*At least validated by a RCT3 for another type of cancer*	2/16; 12,5 %	3/26; 11,5 %
*At least validated by an observational study in another type of cancer*	4/16; 25 %	6/26; 23,1 %
*Other*, *namely never*	2/16; 12,5 %	1/26; 0,04 %
*Other*, *namely descriptive*:	“tissue-based labelling should be changed” (1/16; 6,25 %), “Bayesian approach should be used” (1/16; 6,25 %)	“only as part of a trial” (2/26; 0,08 %) and 7 individual comments (0,04 % each) including “Based on RCTII data”, “Based on RCTII with molecularly selected patients”, “Casuistic evidence from other disease entities”, “Any time”, “tissue-based labelling should be changed”, “Bayesian approach should be used”, “Depending on costs”

Respondents were presented with the following hypothetical situation: “A NGS gene panel was only able to identify one molecular target in a patient’s tumour. However, the corresponding targeted therapy has not been registered for that type of cancer yet, thus off-label treatment may be the only option” They were then asked based on what level of evidence and stage of disease they would prescribe the therapy. The question regarding the metastatic setting was asked in all versions of the questionnaire, while the question for the adjuvant setting was only posed in the physicians and policy version. Therefore, the number of respondents per column differs

Some respondents pointed out that targeted therapies require novel/alternative study designs for evidence generation. Respondents estimated that there is a 65,5 %(±27) chance that revised evidence generation will be generally accepted, as described in scenario 9 (Table 2).

Roughly half of respondents themselves consider evidence from prospective-(46 %) or retrospective observational studies (50 %) valid to base medical decisions on. 14 % chose the option “Lower levels of evidence” and 18 % ticked “Other” which some supplemented with suggestions such as basket-and adaptive protocols as acceptable designs. 72 % of respondents rated the alternative endpoint progression-free-survival valid to base medical decisions upon, compared to 52 % in case of time-to-progression,−41 % in case of disease-free-survival (41 %) and–41 % in case of response rate.

### Market access

Respondents estimated a 45,0 % (±21,7) chance that an alternative technology to NGS will enter the market and decrease NGS popularity, as described in scenario 10. Scenario 11, in which NGS competitors’ propensity to share generated biological insight was described as reluctant, was deemed 45,6 % (±28,7) to occur (Table 2).

Addressing parameters specific to these scenarios, respondents estimated that it will minimally take 6,5 (±6,3) years before NGS gene panels become widely implemented in the clinic and that another technology is likely to take over the market within 9,6 (±5,5) years (Table 3). According to some (60 %), acquired resistance to therapy may be a limiting factor of adoption of NGS. A competing technology will probably still be based on sequencing, but complemented by proteomics/immunotherapy or diagnostics on circulating tumor cells. Novel drug sensitivity assays were also mentioned.

## Discussion

Using scenario drafting on the basis of expert elicitation, we have been able to identify a number of critical factors that may affect the speed of adoption of NGS gene panels in clinical oncology as well as variables that may be incorporated in future cost-effectiveness modeling.

The outlook of patients and physicians towards this novel technology appears to be one of the pivotal facilitators of adoption of NGS panels in the clinic. Although not asked to patients themselves, our results indicated that physicians estimation was that patients’ perspective on NGS panels is likely to be highly positive (~66,5 %), coinciding with studies on pharmacogenomics/-genetics that did include patients in their study population [15, 16]. Popularity amongst patients will probably be positively related to stage of disease, although this distinction seemed related to respondents’ personal opinions. Thus, further investigations into patients’ perspective may be advisable, both in general as well as to confirm the validity of respondents’ estimations. Nonetheless, our results do imply that social factors among patients are likely favorable towards the implementation of NGS panels in the clinic.

We also found that most physicians (~85 %) are probably willing to use NGS panels in clinical practice, given validated evidence from RCTs. Lower levels of evidence are likely to have a large negative impact on adoption rates. Naturally, such stringent levels of evidence come at a cost. However, opting for a prospective observational study compared to a RCT might save time, money and effort, while still convincing the majority (~60 %) of physicians. Importantly, the average physician will require ~25 h of extra training on pharmacogenomics before being able to use NGS panels clinically. Thus, organizing educational activities will definitely be required prior to NGS implementation. Fortuitously, advancing physicians’ knowledge on pharmacogenomics is likely to increase adoption rates even further, as previously stated by Stanek et al. (2012) [17]. Thus, while most physicians stand positive towards NGS, investing in the level of supporting evidence as well as education on NGS panels may even increase the speed of its diffusion.

Next to estimating that the adoption of NGS will benefit from current social perspectives, experts also found it highly feasible that analysis of biopsies can be performed within a clinically relevant timeframe of 10 days. Thus, achievability of timely logistics may also be labeled as a so-called facilitator of diffusion.

However, obtaining a biopsy in itself and whether acquired tissue will meet the criteria for NGS-based analysis may be troublesome on some occasions. While previous investigations have concluded that most European institutes are able to supply FF biopsies meeting standards for RNA/DNA analysis [9, 18], our respondents’ opinions regarding this matter varied extensively. Thus, obtaining and preserving such biopsies may still require a learning curve for a large number of hospitals and could potentially pose a barrier for widespread adoption of NGS panels. The 90,5 % sensitivity, 89,0 % specificity or 18,4 % maximum failure rate described in this article may serve as a useful guideline during development regarding decisions on the trade-off between user-friendliness and publically desired technical specifications and as such, may help in increasing adoption rates.

One of the general barriers for NGS implementation and adoption includes low probability of reimbursement. This could pose a major barrier for implementation, since costs associated with NGS testing − while dropping-and targeted therapies themselves are still not affordable for patients themselves. According to our findings, even combined efforts to promote reimbursement policy are unlikely to succeed (~40,3 %). Thus, the necessity to steer the development of such panels towards the most cost-effective solution, thereby increasing likelihood of reimbursement, is obvious and highlights the potential of early Technology Assessment. Furthermore, the probability to opt for an FFPE-based NGS panel if priced at €1000 was averaged at 44 %. Recently, Kilambi and colleagues (2014) found that the willingness-to-pay (WTP) for accurate test information regarding colorectal cancer screening was approximately $1800 [19]. Thus, it appears that WTP has a wide variance and could be interesting for further research.

Furthermore, demonstrating the effectiveness of guiding therapy via NGS panels also may pose a problem. While respondents expect to identify 6,6 molecular targets per patient, the number of actionable drug targets may actually remain limited. In 30,2 % of cases, off-label prescription will be required, which most physicians would be willing to supply albeit depending on stage of disease and level of evidence. While perhaps helpful in some cases, these situations actually underline the need for accelerated evidence generation on drug efficacy as to increase the number of registered therapies on the market. Almost all of our respondents deemed lower levels of evidence than traditional RCTs valid to base medical decisions upon and without encouragement towards that direction, some even advocated the need for more flexible (eg. basket/adaptive protocols) and molecularly orientated designs. A recent review by Sargent and Korn (2014) affirmed that there has already been a major shift in the paradigm surrounding cancer clinical trial designs in the past decade, in which molecular classification is gaining in popularity [20]. Indeed, this topic is not seldom discussed at scientific-meetings and in literature. Any such efforts are likely to benefit the clinical utility of NGS gene panels as well and may perhaps even be pivotal in their road to success.

Nonetheless, it will take approximately 6,5 years before NGS gene panels become common in clinical oncology and competition on the market can be expected to be fierce. Since our respondents believe that there is a considerable chance (45,0 %) that another novel technology will rapidly become more popular, even within 9,6 years, the window of opportunity for NGS gene panels is small. Thus, timely reaching the market may be crucial for developers. We believe that our findings and suggestions can contribute to that process.

However, our results also face several limitations. Since NGS for diagnostics is rather complex to begin with, some topics such as data warehouse and–integration, were not incorporated in our research yet as they require the in-depth expertise of completely different stakeholders. Some groups, such as patients, clinical geneticists, pharmaceutical companies or health insurers were not included in our study and should be approached in future research. Furthermore, our results are at risk for response bias, since respondents are likely to have an increased interest in NGS a priori. Since we decided not to focus on a particular NGS panel for the generalizability of our results, some questions elicited vastly different opinions or outcomes. Next to the response bias, there might also be a framing bias; a person’s choice between alternatives depends on how these alternatives are framed. Although there has been a pilot series of 6 responders from different disciplines where we tested the wording in case of suggestiveness, it was not completely possible to prevent. In particular, scenarios were framed positively or negatively to evoke the responder to his or her opinion, yet this may have also led to suggestiveness of the response. Large standard deviations due to (un) intentional ambiguity in language or small sample size, disagreement or outliers can make it difficult to draw clear-cut conclusions.

## Conclusions

To our notion, we are the first to methodologically assess NGS, mapping out both quantitative and qualitative aspects that may influence adoption of this novel technology in the clinic.

We believe our findings enable readers involved in NGS implementation to anticipate pivotal events of social, technical, clinical and financial nature (Table 2) and if applicable, alter their strategy to improve success. For instance, by meeting the technical specifications desired by physicians or opting for development of user-friendly FFPE-capable panels. Increasing adoption may also be achieved via hosting education events (Table 5). Perhaps even more important, will be to tackle anticipated barriers for adoption. For instance, further efforts will be required to accelerate, demonstrate and perhaps improve clinical utility of NGS panels (Table 5).

**Table 5:** Recommendations to promote adoption of NGS in clinical oncology

Domain	Recommendation
Social	▪ Further investigate patients’ perspective ▪ Organize additional training on pharmacogenomics for physicians.
Technical	▪ Use desired technical specifications as a guideline for development of a NGS panel. ▪ Develop user-friendly FFPE-capable NGS panels.
Reimbursement	▪ Further investigate willingness-to-pay ▪ Set cost-effectiveness as a high priority to facilitate reimbursement.
Clinical utility and evidence generation	▪ Advocate novel evidence generation designs.
Market access	▪ Enter the market rapidly to maximize window of opportunity

*FFPE* fresh frozen paraffin embedded [tissue preservation]

In addition, our drafted scenarios and estimates on accompanying parameters may be used for (the setup of) cost-effectiveness modeling (Table 3). Such data is a valuable starting-point at this early stage of development, since traditional resources of information are not yet available. Importantly, modeling outcomes alongside development could steer NGS towards the most optimal outcome. As reimbursement probability was also found to be low in our study, we believe such efforts should receive high priority.

To conclude, we have taken the first steps towards scientific input for reimbursement decisions, thereby potentially accelerating the route from bench-to-bedside.

## Additional files

Additional file 1:
**Questionnaire.** Description: This is the full version of the questionnaire, as presented to physicians. Questions also posed in the biologists’ version are marked by “Bio” and questions also posed in the policy version are marked by “Pol“. (PDF 724 kb) Additional file 2:
**Relationship between physicians' opinion on when to offer NGS to patients and their estimations on popularity of NGS among patients.** Description: Depicted results correspond to questions 1 and 7 in the questionnaire (Additional file 1). Respondents’ estimations on popularity of NGS among patients may have been biased by their own views (DOCX 12 kb)

## Abbreviations

CRC: colorectal cancer
FF: fresh frozen
FFPE: formalin-fixed paraffin embedded
HTA: health technology assessment
NGS: next generation sequencing
NKI: Netherlands cancer institute
NSCLC: non-small cell lung cancer
RCT: randomized controlled trial
WTP: willingness-to-pay
