# Supplementary material for: Scenario drafting for early technology assessment of next generation sequencing in clinical oncology
Source: BMC Cancer. 2016 Feb 6;16:66. doi: 10.1186/s12885-016-2100-0 (PMC4744630; doi:10.1186/s12885-016-2100-0)
Supplement: Additional file 1: — Questionnaire. Description: This is the full version of the questionnaire, as presented to physicians. Questions also posed in the biologists’ version are marked by “Bio” and questions also posed in the policy version are marked by “Pol“. (PDF 724 kb) [file 12885_2016_2100_MOESM1_ESM.pdf]

Supplement 1.

This is the full version of the questionnaire, as presented to physicians.

Questions also posed in the biologists' version are marked by "Bio" and questions also posed in the policy version are marked by "Pol".

ANTONI VAN LEEUWENHOEK
NEDERLANDS KANKER INSTITUUT

Dear sir or madam,

Personalized or targeted therapy is believed to improve patient outcomes and reduce adverse effects, especially in oncology. Many institutes are currently developing next generation sequencing (NGS) gene panels, which can identify multiple mutations (=targets) that are specific for a patient's tumor. Subsequently, such a patient receives a targeted therapeutic, such as Erlotinib (targets mutant EGFR), which hopefully results in good clinical outcome. The costs for NGS have dramatically decreased in the past years, which may facilitate the implementation of personalized medicine in the clinic.

Nevertheless, several challenges still lie ahead before such an approach will become standard clinical practice. To anticipate these problems and to model cost-effectiveness of such a NGS gene panel, we ask your expert *estimation* and opinion on several aspects related to the clinical adoption and -dissemination of such panels. We will use your input to draft several scenarios, which will be presented and discussed at a workshop in the near future, to generate consensus.

It is important to note that we focus on NGS gene panels, which are not specific for one type of cancer. Thus, the same panel can be used for several types of cancer. For the moment, we will focus on the use of a NGS gene panel applicable for melanoma, colorectal cancer (CRC) and non-small cell lung cancer (NSCLC).

This questionnaire consists of two parts.

Part one contains 41 questions. If you feel that you cannot possibly answer a certain question, because it is not within your expertise, you can skip that question. Part two consists of a dozen statements, which we ask you to rate.

We estimate that this questionnaire will take less than 15 minutes of your time.

This document is an interactive PDF, you can type and tick boxes instantly!

Thank you very much for your input, time and effort!

Example of workflow

The figure below represents an example of a workflow for analyzing tumor biopsies and determining the best therapeutic. Patient and physicians may choose for the traditional histology based route or for genetic analysis via NGS. This figure is only meant for visual clarification.

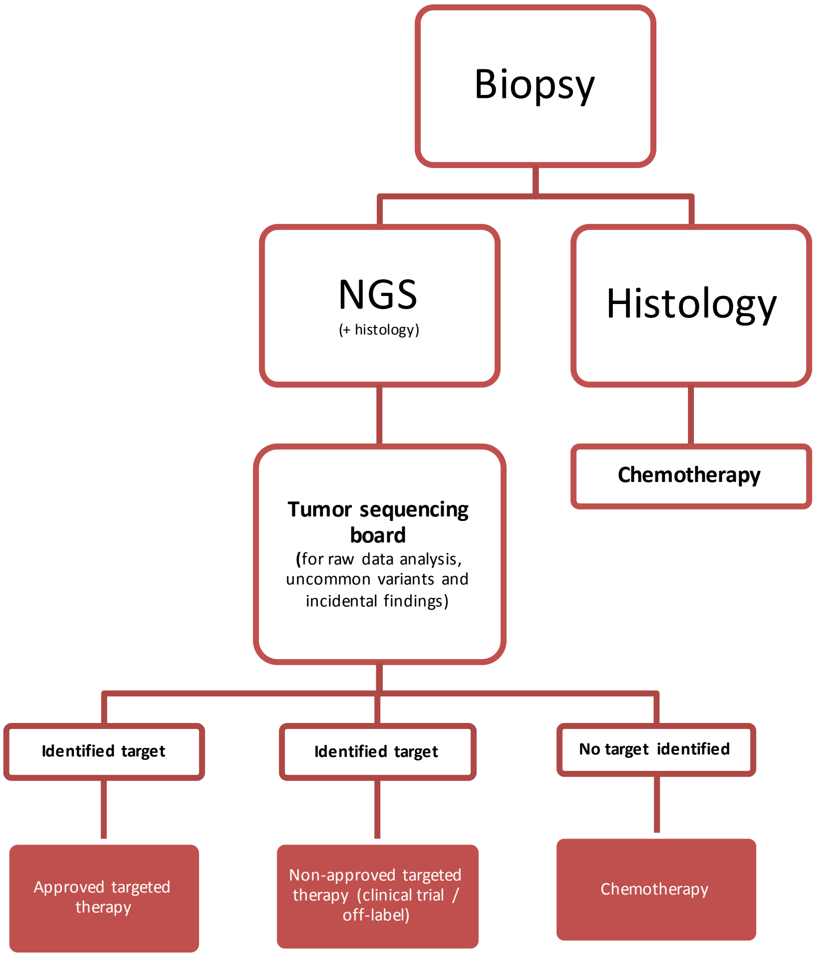

Respondent descriptives

Please state your profession:
Years of professional experience:
Please state the country you work in:
If applicable, which cancer type lies within your specialization?:

Part 1: Parameters

Patient perspective

1. In general, what percentage of cancer patients do you expect to choose for NGS genetic testing of their tumor first, rather than histology + standard chemotherapy?
(Please see the figure above for visual clarification)

% of patients suffering from primary cancer (adjuvant)
% of patients suffering from metastatic cancer

2. Imagine a NGS gene panel identifies only one (potential) therapeutic target in the tumor of a patient. The corresponding targeted therapy might either be FDA approved or is still subject of a clinical study. In the latter case, what percentage of patients do you expect to be willing to participate in a clinical study on the new targeted therapy?
(Please see the figure above for visual clarification)

% of patients suffering from primary cancer (adjuvant)
% of patients suffering from metastatic cancer

3. How much *extra* time do you expect a doctor needs to inform a patient on NGS-based genetic testing, during the first consult explaining this approach?

minutes (0 is possible)

4. Germline mutations not relevant for the treatment of the current cancer may be encountered during genetic testing. Informed consent forms may present different options to patients. Please estimate the percentage of people that will choose for the following options:

____% of patients will indicate that they want to be informed on a germline mutation that predisposes them to a disease that can be prevented or treated.

____% of patients will indicate that they want to be informed on a germline mutation that predisposes them to a disease that cannot be prevented or treated.

____% of patients will indicate that they want to be informed on a germline mutation that does not affect their own health, but may be important for the health of relatives.

____% of patients will indicate that they want to be informed on uncertain germline mutation that may or may not be important to their own health or that of relatives.

____% of patients will indicate that they do not want to be informed on any germline mutations at all.

Professional's perspective

5. At what level of evidence would you be willing to use (or allow the use of) NGS-based sequencing results in clinical practice, generated using a NGS panel? (Pol)

   If at least validated by randomized controlled trials (RCT) ☐
   If at least validated by prospective observational studies ☐
   If at least validated by retrospective observational studies ☐
   If at least validated by lower levels of evidence ☐

6. What percentage of physicians, do you estimate, are willing to use such validated NGS gene panels?

   If at least validated by randomized controlled trials (RCT): %
   If at least validated by prospective observational studies: %
   If at least validated by retrospective observational studies: %
   If at least validated by lower levels of evidence: %

7. At what stage of disease or point of treatment should patients be offered a NGS gene panel? (Pol)

8. How many years will it at least take before patients are offered a NGS gene panel for adjuvant treatment? (Pol)

   years

9. Stakeholders will use NGS gene panels in clinical practice, provided the result of a biopsy is returned within maximally ___ days.

10. Raw NGS results are difficult to interpret; a team of experts will analyze the information and present an advice to the physician. How much extra education will the average clinical oncologist (situated in a general hospital) require to start using the test and adequately inform patients? (Bio)

   Average of ___ hours

Cost-effectiveness

11. Please rank the three most important criteria that, according to you, should be considered when determining which genes should be included in a NGS gene panel? (Pol, Bio)

Target Prevalence
Effectiveness of associated treatment
Level of evidence
Effectiveness of alternative treatments
Other, namely:

12. Imagine a NGS gene panel that has been clinically validated and sequences 200 genes. How much would you be willing to pay extra for a NGS gene panel, compared to the price of sequencing one single gene, given the additional information it may generate? (Pol)

euro

13. How long should sequencing results, not relevant for the current treatment, minimally be stored? (Pol, Bio)

years

14. How long should excess tumor tissue minimally be stored? (Pol, Bio)

years

Technical aspects

15. What is the minimal sensitivity and specificity (for accurately detecting a mutation) you would demand before considering using (or allow the use of) a NGS gene panel in clinic practice? (Pol)

    Sensitivity   Specificity

16. For technical reasons, FF tissue is preferred over FFPE for NGS-based sequencing. What percentage of institutes within the Netherlands is capable of supplying FF tissue? (Bio)

    % Institutes

17. Biopsies sometimes only contain a small amount of tumor cells/DNA and may not always qualify for NGS. What failure rate would you maximally find acceptable? (Bio)

    Maximally   % of tumor biopsies ineligible for NGS

18. If re-biopsy would be required, what percentage of patients do you expect to decline?

    % in CRC
    % in NSCLC
    % in melanoma

19. If re-biopsy would be required, in what percentage of patients would you consider this non-feasible?

    % in CRC
    % in NSCLC
    % in melanoma

Evidence generation

20. Performing an RCT III for each gene target and therapy for every separate type of cancer, is costly and will take a long time. Thus, such studies may therefore delay the implementation of targeted therapy in the clinic. What other type of study designs would you consider valid to base medical decisions on? (Pol, Bio)

- ☐ Prospective observational study
- ☐ Retrospective observational study
- ☐ Lower levels of evidence
- ☐ Other, namely:

21. What alternative endpoints of clinical studies, rather than overall survival (OS), would you consider valid outcome measures to base clinical decisions on? (Pol, Bio)

- ☐ Progression Free Survival (PFS)
- ☐ Time-to-progression (TTP)
- ☐ Disease Free Survival (DFS)
- ☐ Response Rate (RR)
- ☐ Other, namely:

Imagine that a targeted therapy has been approved for the treatment of melanoma with a mutation in gene X. However, using an NGS-panel, you might encounter mutation X in a biopsy of a patient with metastatic colorectal cancer as well. Due to regulation, you cannot simply prescribe the targeted therapy to this patient, because it has not been approved for use in colorectal cancer.

22. How often do you expect to run into this kind of situation, using NGS-based panels? (Bio)

In   % of the time using a NGS gene panel

23. In such a situation, a physician might choose to still prescribe the targeted therapy, in experimental setting. We would refer to that as off-label treatment. What percentage of physicians would be willing to do so?

% of physicians

24. Based on what level of evidence on a target/therapy combination, would you consider (or allow the use of) off-label treatment in adjuvant setting? (Pol)

- ◯ At least validated by an RCT III in another type of cancer *and* an observational study for the cancer you are dealing with.
- ◯ At least validated by a RCT III for another type of cancer
- ◯ At least validated by an observational study in another type of cancer
- ◯ Other, namely:

25. Based on what level of evidence on a target/therapy combination, would you consider (or allow the use of) off-label treatment in a patient with no further treatment options? (Pol)

- ◯ At least validated by an RCT III in another type of cancer *and* an observational study for the cancer you are dealing with.
- ◯ At least validated by a RCT III for another type of cancer
- ◯ At least validated by an observational study in another type of cancer
- ◯ Other, namely:

26. How long will the medical community as a whole (physicians, insurers, government etc.) minimally need before they become more lenient towards off-label treatment? (Pol)

years

27. What is the probability that health insurers will reimburse off-label treatment? (Pol)

p= (0-1)

28. For the three situations below, please estimate the **additional** progression free survival (PFS) or disease free survival (DFS) that could be gained compared to standard treatment, due to the use of *off-label* targeted therapy based on NGS results.

A patient suffering from metastatic HER2-mutated NSCLC, receiving targeted agent Lapatinib (Tyverb).
Estimated additional PFS: months

A patient suffering from metastatic C-KIT-mutated NSCLC, receiving targeted agent Imatinib (Gleevec)?
Estimated additional PFS: months

A patient suffering from metastatic C-KIT-mutated melanoma, receiving targeted agent Imatinib (Gleevec).
Estimated additional PFS: months

A patient suffering from metastatic BRAF-mutated CRC, receiving targeted agent Dabrafenib
Estimated additional DFS: months

29. How many registered targeted therapies, for new targets, will enter the market within the next five years? (Bio)

targeted therapies

Reimbursement policy

30. Competitors producing NGS-based panels may choose to work on some sort of consensus agreement, which may possibly help in reimbursement policy. Please rank the three most important aspects competitors or other stakeholders should be willing to agree upon, for this to work. (Pol)

Minimal sensitivity/specificity
A certain number of genes
Minimal level of evidence regarding target/treatment combinations
Sharing biological information
Consent forms
Other, namely:

Procedures after finding a germline mutation, such as additional tests or intervention, may result in extra costs. For example, regular colonoscopy may be performed in a patient identified with a higher risk of CRC.

31. What is the probability that patients will be overtreated for other diseases than the current cancer, due to these germline findings? (Pol)

p = (0-1)

32. Some may argue that acting upon early detection of some diseases may result in better clinical outcome. What is the probability that medically acting upon these germline findings will substantially improve the clinical outcome of such a patient? (Pol)

p = (0-1)

Market Success

33. How many years do you estimate, will it take before use of NGS-panels will become quite common in the clinic? (Bio)

years

34. Will the possibility of acquired resistance of the tumor to targeted therapy slow down the adoption of NGS-panels?

○ No ○ Yes

35. Within what minimum timespan do you expect a *different* technology than NGS to outcompete NGS gene panels? (Bio)

years

36. What other technology will most likely do so? (Bio)

NKI specific

The Netherlands Cancer Institute (NKI) is a mid-sized hospital in the Netherlands, specialized in oncology. *Both hospital-employed as well as other Dutch physicians can request tumor sequencing.* The NKI aims to develop a NGS gene panel that includes 178 genes, also sequences RNA (some expression and identify translocations) and is able to handle FFPE-preserved biopsies. Initially, this panel will be used for patients suffering from non-small-cell lung cancer (NSCLC), colorectal cancer (CRC) and melanoma.

37. What number of therapeutic targets, on and off-label, would you expect to find in an average patient with metastatic- CRC, melanoma or NCSLC, using such a large panel? (Bio)

genes

38. Compared to competing NGS gene panels, the NKI panel is quite large, can handle FFPE biopsies and also sequences RNA. What percentage of NGS applicants will choose the NKI panel over competitors, due to these additional features?

%

39. How many requests for NGS do you expect this hospital to receive within seven days?

requests

40. What is the probability that you would use the NKI panel if it was priced at €1000? (Pol)

p= (0-1)

41. What is the maximum delay the NKI panel can have entering the market compared to competitors, before you would lose interest?

months

Part 2: Scenarios

Below you will find statements describing scenarios that may influence the adoption of NGS-panels by the Dutch healthcare market. Please estimate the probability that these scenarios will occur within 5-10 years. The questions you have answered so far, will be used to improve these scenarios.

1. **Patient perspective**
   Patients will demand lots of information on NGS-based panels, but will nevertheless be very interested in using them.
   Estimated probability of this scenario: %

2. **Medical professional perspective**
   Medical professionals remain unconvinced of the clinical benefit that can be gained using NGS-panels and targeted therapy.
   Estimated probability of this scenario: %

3. **Efficiency (Bio)**
   The time required for preparation, NGS and analysis of a biopsy will decrease so that patients will receive results within ten days after biopsy.
   Estimated probability of this scenario: %

4. **FF versus FFPE (Bio)**
   If reliable sequencing results can only be obtained by using FF tissue, the use of NGS-based panels will remain limited.
   Estimated probability of this scenario: %

5. **Clinical Utility (Bio)**
   Demonstrating clinical utility of NGS-panels will take at least a couple more years, adoption of this technology will only succeed once that point is reached.
   Estimated probability of this scenario: %

6. **Revised evidence generation (Pol, Bio)**
   Evidence from other clinical study designs than RCT III, will be considered valid to include new targets in NGS gene panels.
   Estimated probability of this scenario: %

7. **Actionable targets (Bio)**
   The number of mutations identified by NGS panels that can actually be targeted by therapy remains limited.
   Estimated probability of this scenario: %

8. Off-label prescription (Pol)
The medical community as a whole (physicians, government, health insurers etc.) becomes more lenient towards off-label treatment.
Estimated probability of this scenario: %

9. Consensus-gene panel (Pol)
A 'minimal requirements' agreement between institutes developing NGS-based gene panels has resulted in national reimbursement policy of such panels.
Estimated probability of this scenario: %

10. Intellectual property (Bio)
Competitors offering NGS-based panels will be reluctant to share new biological insights generated by NGS-panels with each other, thereby decelerating the improvement of clinical utility for patients.
Estimated probability of this scenario: %

11. Competition within the field (NKI specific)
Another NGS-based panel outcompetes the NKI-panel, regardless of its additional features.
Estimated probability of this scenario: %

12. Competition from another field (Bio)
Another type of technology enters the Dutch healthcare, decreasing the popularity of NGS-based gene panels.
Estimated probability of this scenario: %

Questionnaire improvement

How long did it approximately take you to fill in this questionnaire? **(Pol, Bio)**

minutes

Thank you!

SUBMIT

* This button may not work properly if you have no default email client installed.
Our apologies! If so, please email this form manually to s.joosten@nki.nl
