# Supplementary material for: Scenario drafting for early technology assessment of next generation sequencing in clinical oncology
Source: BMC Cancer. 2016 Feb 6;16:66. doi: 10.1186/s12885-016-2100-0 (PMC4744630; doi:10.1186/s12885-016-2100-0)
Supplement: Additional file 2: — Relationship between physicians' opinion on when to offer NGS to patients and their estimations on popularity of NGS among patients. Description: Depicted results correspond to questions 1 and 7 in the questionnaire (Additional file 1). Respondents’ estimations on popularity of NGS among patients may have been biased by their own views (DOCX 12 kb) [file 12885_2016_2100_MOESM2_ESM.docx]

|  | | **Patient popularity estimations (Q1)** | | |
| --- | --- | --- | --- | --- |
| **Physicians’ own opinion (Q7)** |  | **Primary** | **Metastatic** | **Δ Estimations** |
|  | **Primary (n=6)** | 35,0 (±26,7) | 69,0 (±27,8) | 34,0 (±24,5) |
|  | **Metastatic (n=6)** | 28,5 (±36,4) | 72,5 (±37,1) | 44,0 (±34,0) |
